# Projecting the impact of Covid-19 variants and vaccination strategies in disease transmission using a multilayer network model in Costa Rica

**DOI:** 10.1038/s41598-022-06236-1

**Published:** 2022-02-10

**Authors:** Yury E. García, Gustavo Mery, Paola Vásquez, Juan G. Calvo, Luis A. Barboza, Tania Rivas, Fabio Sanchez

**Affiliations:** 1 Department of Public Health Sciences, University of California Davis, Davis, CA 95616 USA; 2 Centro de Investigación en Matemática Pura y Aplicada, Universidad de Costa Rica, San José, 11501 Costa Rica; 3 Pan American Health Organization, World Health Organization, San José, 10102 Costa Rica; 4 Centro de Investigación en Matemática Pura y Aplicada-Escuela de Matemática, Universidad de Costa Rica, San José, 11501 Costa Rica; 5 Ministry of Health, San José, 10102 Costa Rica

**Keywords:** Diseases, Health care, Mathematics and computing

## Abstract

For countries starting to receive steady supplies of vaccines against SARS-CoV-2, the course of Covid-19 for the following months will be determined by the emergence of new variants and successful roll-out of vaccination campaigns. To anticipate this scenario, we used a multilayer network model developed to forecast the transmission dynamics of Covid-19 in Costa Rica, and to estimate the impact of the introduction of the Delta variant in the country, under two plausible vaccination scenarios, one sustaining Costa Rica’s July 2021 vaccination pace of 30,000 doses per day and with high acceptance from the population and another with declining vaccination pace to 13,000 doses per day and with lower acceptance. Results suggest that the introduction and gradual dominance of the Delta variant would increase Covid-19 hospitalizations and ICU admissions by $35\%$ and $33.25\%$, respectively, from August 2021 to December 2021, depending on vaccine administration and acceptance. In the presence of the Delta variant, new Covid-19 hospitalizations and ICU admissions are estimated to increase around $24.26\%$ and $27.19\%$, respectively, in the same period if the vaccination pace drops. Our results can help decision-makers better prepare for the Covid-19 pandemic in the months to come.

## Introduction

In the fight against the Covid-19 pandemic, the remarkable time frame at which scientists achieved to develop safe and effective vaccines^1,2^ has provided a crucial tool in the global effort to reduce the widespread health, economic, and social disruption caused by the emergence of the severe acute respiratory syndrome coronavirus 2 (SARS-CoV-2). As of July 2021, just 18 months after the genetic sequencing of SARS-CoV-2^3^, a total of 21 vaccines have been granted emergency use authorization by countries around the world^4^, while 108 vaccine candidates are at different phases of clinical development^5^.

Despite these extraordinary achievements, worldwide vaccination programs are facing challenges, such as supply shortages^6^, mainly in low-to-middle-income countries (LMIC)^7^, and low acceptance rates, mostly in their high-income counterparts^8,9^.

Furthermore, the world is facing the ominous threats of rapidly appearing SARS-CoV-2 variants^10–12^. Several variants of concern have already increased the transmissibility of the virus^13^ and the severity of cases among younger population groups^14,15^. Moreover, these variants have shown evidence of reduced effectiveness of current vaccines, at least in lowering disease transmission^16,17^. We have already started to hear calls for third doses to fight more aggressive variants^18^, which are more likely to appear if vaccine supplies remain scarce in large parts of the world.

In light of this, unless worldwide vaccine distribution becomes more homogeneous, the current vaccine shortages in large parts of the world will perpetuate SARS-CoV-2 circulation, which ensures the relentless appearance of new variants^19^. Something similar is expected in countries where, despite having enough vaccines, large pockets of the population remain unvaccinated due in part to misinformation and ineffective health promotion and communication strategies^20,21^.

Under this context, the development of new models is providing health authorities and decision-makers around the world an evidence-based tool to inform, guide, update and adapt public health interventions to their unique circumstances and available resources. These models can be used as a tool to forecast potential transmission scenarios and the associated healthcare demand under different vaccination scenarios^22–29^ and circulating variants^30,31^.

In line with these efforts, the current study was developed by implementing a tailored multilayer network model for Covid-19 in Costa Rica. The objective of the analysis was to project and assess the impact of the introduction of the more transmissible B.1.617.2 (Delta) variant in the country. We compared the effects of two different vaccine administration scenarios on the number of SARS-CoV-2 infections, deaths, and Covid-19 related hospital admissions from August 2021 to December 2021. We were also able to estimate the direct base cost of providing hospital care. Since vaccination pace depends on the availability of the vaccine and the acceptance by the population, vaccination scenarios in the model considered both the speed of vaccine administration and percentages of vaccine hesitancy from the population.

This study considered the information available by July 31, 2021. As of that date, Costa Rica had only administered the Pfizer-BioNTech (BNT162B2) and AstraZeneca (ChAdOx1-s) vaccines. The vaccination program in the country began on December 24, 2020. For its distribution, health authorities notified a vaccination strategy which divided the population into five groups taking into consideration the age of the population, work-related risk of exposure and preexisting medical conditions. These groups and their opportunity to receive the jabs have been adjusted and revised frequently by the National Vaccination Committee as new evidence and the supply of vaccines became accessible^32^. By May 18, 2021, the majority of the vaccinated population had received the 21-days interval scheme with BNT162B2, as the first ChAdOx1-s vaccines entered the country during the second week of April. After evidence of increasing the intervals between doses arose^33^ the government announced a 12-week interval between doses for both the BNT162B2 and ChAd0x1-s vaccines for people under 57 years of age. Thus, we considered that all vaccinated individuals received their second dose 12 weeks after the first dose as of May 18, 2021. By July 20 2021, the country had increased its vaccination capacity and it was inoculating at a rate of 30,000 doses per day, reaching $38.4\%$ of the total population covered with one dose and $16.1\%$ fully vaccinated by that same date. However, several cantons in the national territory were showing lower vaccination acceptance, and vaccine hesitation appear higher among younger and healthier individuals, which threatens to mirror declines in vaccination rates observed in several other countries.

On the other hand, by July the country was facing the imminent arrival of the most transmissible Delta variant. Earlier in 2021, the country saw its worse Covid-19 crisis between May and early June, period that coincides with the introduction of the Alpha and even more dominant Gamma variants. Cases by late June and early July were rapidly dropping at last, as Covid-19-related hospitalizations and deaths were too. But countries around the world were facing rapid resurgences in cases and hospital admission due to the newest Delta variant^34^. It was uncertain how the introduction of this more aggressive pathogen will affect the pandemic in the country by the end of 2021, when vaccination coverage was expected to reach numbers which were once thought to provide herd immunity.

## Methods

### Data

Publicly available information of Covid-19 regarding daily reported cases, hospitalization, and ICU admissions was obtained from the official website of the Ministry of Health of Costa Rica^32^. We used demographic data of the 5,163,038 inhabitants of the country to establish the contact network. This data included population by age, canton of residence, and workplace, as reported by the National Institute of Statistics and Census (INEC)^35^. All the methods were performed in accordance with relevant guidelines and regulations.

For the initial conditions of the model, we incorporated the number of confirmed Covid-19 cases from March 6, 2020 (day the first case was reported in the country^32^), to June 28, 2021, including the age and canton of residence of the patients as notified by the health authorities. During this period, the cumulative number of detected cases ascended to 403,511 with a case fatality rate of $1.3\%$^32^. Parameters related to hospitalization that involve duration of stay, percentage of hospital admissions by age group, and hospitalization costs were provided by the Social Security Fund of Costa Rica (CCSS), public entity in charge of both general and specialized medical care for the Costa Rican population. According to data from CCSS, the estimated base cost of hospital bed per day, (which doesn’t include diagnosis procedures or treatment) is approximately 707,540 colons (1,137 USD) in the medical ward and 902,641 colons (1,450 USD) in an ICU^36^. It is worth stressing that hospital charges in reality are substantially higher for Covid-19 patients when all procedures, specialist care and exams are considered, and that cost varies with length of stay.

We also used the weekly number of administered Covid-19 vaccines as reported by CCSS from December 24, 2020 to July 28, 2021^37^. According to health authorities, the target population to be vaccinated in Costa Rica is 4,274,344, a $83\%$ of the total population which includes all residents that are 12 years and older. During this period, $55\%$ of the target population had received the first dose, while $19\%$ received the second dose.

As for the percentage of vaccine hesitance, we took into account the available information of administrated vaccines by age group as of July 2021 in Costa Rica, as well as information reported from previous studies, where a median of vaccine acceptance of $78\%$ has been observed in LMICs^38^.

### Model

We extended a multilayer, temporal and stochastic *network model* first developed to simulate the spread of the SARS-CoV-2 in Costa Rica. A complete description and implementation is presented in^39^. The scenarios were constructed to include different vaccine administration speeds, the introduction of the Delta variant, and population resistance to available Covid-19 vaccines in the country.

The multilayer structure of the model incorporates three layers representing people living in the same house, friends or coworkers, and sporadic contacts. The probability of transmission in each layer is different, and while the contacts of the household layer remain fixed, the other two layers have variations according to public health measures and changes in social behaviour. The model contemplates ten mutually exclusive compartments: susceptible, latent, diagnosed (infectious) and undiagnosed (infectious), hospitalized, individuals admitted to intensive care, recovered, successfully vaccinated with one dose, fully vaccinated, and Covid-19 deceased individuals.

To include the difference in disease severity among age groups^40^, vaccination strategies implemented by the Costa Rican health officials, and vaccine hesitance, the model is divided into three age classes: children (0-18 years), adults (19-64 years), and elderly people (+65 years). Assumptions related to disease progression and transmission characteristics remain the same as the original model^39^. However, new hypotheses were introduced to forecast the effects of the different vaccination scenarios.

Individuals to be vaccinated are randomly chosen from the susceptible and recovered compartments. From December 24, 2020, to April 22, 2021, the model incorporated the condition that all recovered individuals would be vaccinated 90 days after their recovery. However, starting from April 23, 2021, this assumption was changed, after health authorities announced that individuals who have already been infected could be vaccinated regardless of the time since their recovery^32^.

Given the available evidence, the model takes into account vaccine effectiveness for the Pfizer-BioNTech and AstraZeneca vaccines. Studies have shown that after the first dose with either of these two vaccines (pre-introduction of the Delta variant), protection against symptomatic illness ranges from $55\%$-$70\%$, while vaccine effectiveness against hospitalization has been reported to be $75\%$-$85\%$^41,42^. Given these levels of protection, our model does not differentiate between administered vaccines. As for vaccine effectiveness against the Delta variant, we assumed a reduction in protection against symptomatic disease after both first and second dose as reported in^16,42^.

Simulations were performed in Matlab R2020a ^43^. We used two remote servers: (i) a Dell PowerEdge R740 with 64GB of RAM and two Intel®Xeon®Silver 4114 CPU @ 2.20GHz processors, and (ii) a Lenovo SR650, with two Intel Xeon Plata 4214, 2.20 GHz processors with 128GB of RAM.

### Scenarios

We developed two scenarios considering the maximum and minimum average of daily first doses during the initial seven months of the vaccination campaign in Costa Rica. The first vaccine scenario contemplates an accelerated vaccination rate with 30,000 daily doses and a high percentage of acceptance by the population, $85\%$ for those aged between 19 and 64 and $95\%$ for people older than 65. The second scenario consists of having a decelerated vaccination rate with 13,000 daily doses and a low acceptance by individuals to receive the vaccine, $70\%$ for those aged between 19 and 64 and $90\%$ for people over 65 years old.

Vaccine administration speed is implemented in two contexts: when circulating variants up to the first half of 2021 continue to dominate for the remainder of the year, and when the Delta variant is detected and gradually becomes dominant.

For the case where the Delta variant is detected during mid-July and becomes dominant two months after, we assumed a vaccine efficacy against symptomatic infections to be $55\%$ at the end of August and $45\%$ from mid-October for those with a single dose. It is important to note, that these percentages consider that other variants will continue to circulate; therefore, we did not decrease vaccine protection against symptomatic illness for the Delta variant to the $30\%$-$35\%$ reported in literature^16,42^. For those fully vaccinated, these percentages are $82\%$ at the end of August and $72\%$ from mid-October onward. No changes are made in the percentages of hospitalization. Recent studies showed that two doses of Pfizer-BioNTech (BNT162B2) or AstraZeneca (ChAdOx1-s) have similar effectiveness in preventing hospitalizations for the Delta coronavirus variant as they are against the previously dominant variants^16,42^.

Vaccines are also administrated according to the age of the population, starting with people over 65 years old, then people between 19 and 64, and finally between 12 and 18. Initially, $85\%$ of the total doses were given to people over 65 years old and $15\%$ to people aged 19 to 64 years. When the target percentage of people in each age group is reached, $100\%$ of the vaccines are administrated in the next available age group. All simulations assumed an efficacy against symptomatic infection of $63\%$ after the first dose and $90\%$ after the second one. These percentages change over time in the scenarios considering the introduction of the Delta variant.

Values reported in the literature suggest protection against hospitalization ranging between 75% and 99% for those with one or two doses ^17,41,50,51^. Therefore, to simulate the increase in the protection conferred by the second dose, we increased protection against hospitalization in the model as the fully vaccinated target population increases. We assumed $80\%$ protection against hospitalization for people vaccinated with one or two doses until $40\%$ of the target population is fully vaccinated, $85\%$ of hospital protection until $60\%$ of the population has a complete scheme, and $90\%$ protection when more than $60\%$ of the target population completes two doses. The percentages remain within the values reported in the literature.

As vaccination rates increase, we assumed that people begin to feel safe, decreasing the percentage of people adhering to non-pharmaceutical interventions (NPI).

We incorporated two parameters in the model to simulate individuals following social distancing measures and those who implement individual protective measures such as using a mask and washing hands: $\%$ of human mobility and $\%$ of individuals following NPI. We sampled the values from a uniform distribution for each simulation. The initial values for human mobility $\%$ range from $60\%$ to $70\%$. Those limits were changed on July 12, when schools opened. The initial values for the $\%$ of individuals following NPI range from $65\%$ to $80\%$. Values for both parameters are modified when $60\%$ of the target population is fully vaccinated. The flexibility of sanitary measures, pandemic fatigue, and the feeling of security are emulated through these parameters. All scenarios are simulated until December 31, 2021. Parameter values and a summary of the assumptions are presented in Table 1.

Results are presented by taking two approaches. First, we compared variation in cases, deaths, and hospitalizations when the vaccination assumptions changed and the variants in circulation are the same by the rest of the year (scenario 1 and 2 in Table 1, with Delta and current dominant variants). Then, we compared the impact of the introduction of the Delta variant with different vaccine coverage compared to the trends without the Delta variant. We also estimated the average base costs of the hospital stay in each scenario.

**Table 1:** Summary of scenarios.

Parameter	Scenario 1	Scenario 2
Daily doses from August 2, 2021	13,000	30,000
% individuals over 65 that are not vaccinated	10	5
% individuals from 19 to 64 that are not vaccinated	30	15
% human mobility	From July 12, 2021, a random value is chosen between 55% and 70%.	
	Then, when 60% of the target population is fully vaccinated the value is chosen from 40% to 55%.	
% individuals following $\hbox {NPI}^*$	A random value is chosen between 50% and 70% when 60% of the target population is fully vaccinated.	

Parameter	Before delta	Delta variant
% protection against symptomatic infection, first dose	63	55 after August 30, 2021
		45 after October 15, 2021
% protection against symptomatic infection, second dose	90	82 after August 30, 2021
		72 after October 15, 2021

$^*$Personal protection measures such as mask use, washing hands and keeping social distance.

## Results

**Figure 1:**
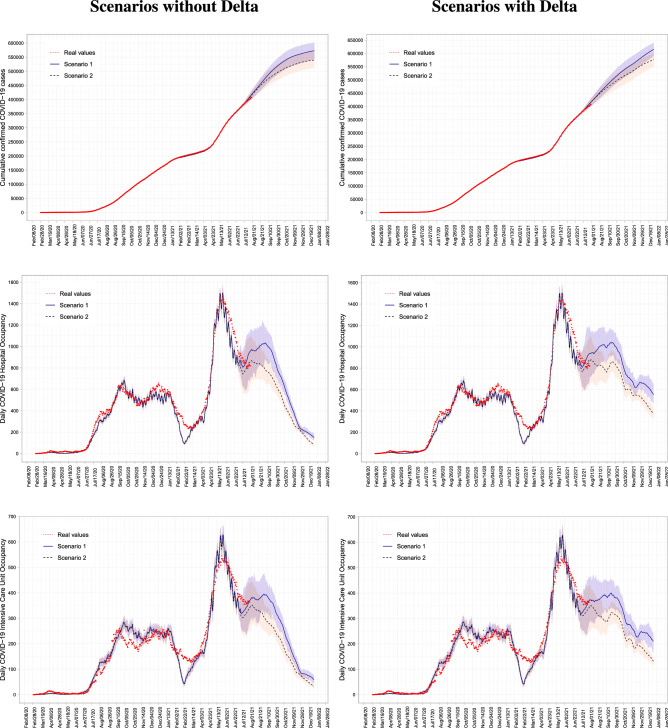
Scenarios without and with Delta Variant. Figures show the scenarios implemented for Costa Rica with 5 million inhabitants, considering two vaccination strategies with and without the presence of the Delta variant. Left panels correspond to the scenario without the introduction of Delta, and the right panels correspond to the results with the Delta variant. From top to bottom, cumulative Covid-19 confirmed cases, daily Covid-19 hospitalizations, and ICU Covid-19 occupancy. Projections are for the period from August 2 to December 31, 2021.

The results correspond to the average of 50 simulations with a $95\%$ confidence interval (CI). The outcome of our simulations is illustrated in Fig. 1 and all results are summarised in Table 2.

Among many others, low vaccine coverage translates into an economic impact for the hospital care system. According to data from the CCSS, in the country, Covid-19 patients are hospitalized from one to fifteen days in the medical ward and five to ten days in the ICU. This length of stay varies by age group. Considering the base cost of hospitalizations in CCSS and an average stay of 10 days in the ward, the cost of a Covid-19 patient for the public health system of Costa Rica would be around 11,370 USD.

Likewise, if patients are admitted to intensive care units for eight days on average, a patient would cost 11,600 USD, both without considering exams or procedures. We present the aggregated cost for basic hospital care taking these values into account.

### Scenario 1 and 2 considering the current variants as dominant for the rest of the year

A daily reduction in the administration of vaccines from 30,000 to 13,000 doses could lead to an increase in the cumulative confirmed cases by $6.20\%$ and total deaths by $7.9\%$ at the end of the year. Hospitalizations and ICU admissions from August 2 to December 31, 2021, could increase by $25.87\%$ and $28.30\%$ respectively ( from an average of 8,982 to an average of 11,306 for patients from the Covid-19 room and from 2,947 to 3,781 for ICU admissions). Low vaccine coverage may increase the country's hospital stay costs by 26,423,880 USD in the ward and 9,674,400 USD in ICU from August 2 to December 31, 2021.

### Scenario 1 and 2 considering that Delta variant gradually becomes dominant over time

When we consider the circulation of the Delta variant and a reduction in daily vaccine administration, cumulative confirmed cases and cumulative deaths could increase by $6.75\%$ and $8.06\%$, respectively, for December 31, 2021. From August 2, 2021, to December 31, 2021, the new hospitalizations and ICUs admissions could rise by $22.66\%$ and $26.08\%$ (from 3,961 to 4,994 hospitalizations and from 7,762 to 7,183 ICU admissions), which in terms of hospital stay expenses correspond to an average increase of 31,654,080 USD in the ward and 11,982,800 USD in ICU.

### Impact of the introduction of the Delta variant with different vaccine administration

We compared the trends with low acceptance and administration of the vaccine doses with and without the circulation of the Delta variant. We projected that the introduction of the new variant might increase the cumulative confirmed cases by $7.43\%$ and the total deaths by $9.04\%$ by the end of the year (from 572,955 to 615,574 cases and 7,095 to 7,762 deaths). From August 2 to December 21, 2021, the cumulative hospitalizations and ICU admissions are projected to increase by $33.30\%$ and $32.08\%$, respectively, representing a cost increment in ward hospitalizations of 42,808,050 USD and 14,070,800 USD in ICU based on a medical ward and ICU costs.

The same analysis in the scenario of high acceptance and vaccine administration leads to an increment of $6.89\%$ in cumulative confirmed cases, $9.3\%$ in cumulative deaths, $36.79\%$ in new hospitalizations (from 8,982 to 12,287), and $34.41\%$ in ICU admissions (from 2,947 to 3,961) representing an average increase in room and ICU stays of 37,577,850 USD and 11,762,400 USD, respectively.

The four scenarios showed an increase in hospital care demand by the end of June. This increment is regardless the introduction of Delta variant, as parameters that simulate its introduction are unchanged until the end of August. The increase in hospitalizations and ICU admissions reach a plateau that decreases slowly with the progressive dominance of the Delta variant.

Our model projected that while maintaining a 12 week-interval between doses, a vaccine rollout of 30,000 doses per day and high vaccine acceptance by the Costa Rica population, around $84.5\%$ of the target population would be vaccinated with at least one dose by mid-September, and approximately $75\%$ would be fully vaccinated by the end of the year. On the other hand, for a daily average vaccination of 13,000 doses per day with low acceptance of the vaccine, approximately $73.33\%$ of the target population would be vaccinated by mid-September, and $66.71\%$ would be fully vaccinated by the end of the year.

**Table 2:** Summary of results.

Description	Current variants			Delta variant			Increase with the introduction of Delta	
	Vaccine strategy 1 13,000 doses per day (95% CI)	Vaccine strategy 2 30,000 doses per day (95% CI)	Increase when vaccination rate is reduced	Vaccine strategy 1 13,000 doses per day (95% CI)	Vaccine strategy 2 30,000 doses per day (95% CI)	Increase when vaccination rate is reduced	Vaccine Strategy 1	Vaccine Strategy 2
Total positive cases by Dec 31, 2021	572,955 (541337, 601308)	539,484 (511916, 569817)	6.20%	615,574 (589813, 640475)	576,657 (550154, 599234)	6.75%	7.43%	6.89%
New hospitalizations from Aug 2 to Dec 31, 2021	11,306 (11318, 14189)	8,982 (6815, 11393)	25.87%	15,071 (11756, 18532)	12,287 (9586, 15092)	22.66%	33.30%	36.79%
New ICU admissions from Aug 2 to Dec 31, 2021	3,781 (2542, 5092)	2,947 (1925, 4139)	28.30%	4,994 (3 495, 6 593)	3,961 (2691, 5336)	26.08%	32.08%	34.41%
Total Deaths by Dec 31, 2021	7,095 (6 604,7 566)	6,572 (6273, 6936)	7.9%	7,762 (7299, 8162)	7,183 (6847, 7567)	8.06%	9.4%	9.3%
Average hospital charge in ward (10 days average stay)	128,549,220 USD	102,125,340 USD	26,423,880 USD	171,357,270 USD	139,703,190 USD	31,654,080 USD	42,808,050 USD	37,577,850 USD
Average hospital charge in ICU (8-days average stay)	43,859,600 USD	34,185,200 USD	9,674,400 USD	57,930,400 USD	45,947,600 USD	11,982,800 USD	14,070,800 USD	11,762,400 USD
Percentage of target people with at least one dose	73.33% (73.21, 73.45)	84.50% (84.35, 84.65)		72.89% (72.74, 73.02)	84.50% (84.37, 84.66)			
Percentage of target people fully vaccinated	66.71% (65.92, 67.53)	76.78% (75.69, 77.80)		65.67% (64.8, 66.57)	75.04% (74.02, 76.26)			

The values and percentages correspond to the results of two vaccine strategies with and without introducing the Delta variant in Costa Rica, a country with five million inhabitants. Hospital expenses are calculated based on a stay of 10 days in the medical ward and eight days in the ICU with an average cost per day of 707,540 colones (about 1,137 USD) in the medical ward and 902,641 colones (about 1,450 USD) in the ICU.

## Discussion

With the emergence of SARS-CoV-2 variants influencing the transmissibility of the virus^11,44^, severity of the disease in younger population groups and the performance of available vaccines^16^, assessing probable future transmission scenarios and its resulting impact on health-care systems is at the forefront of health authorities and decision-makers around the world. By implementing a multilayer network model that simulates the introduction of the Delta variant in Costa Rica under two different scenarios of vaccine administration speed and population hesitance, we pursue to advocate in these efforts and generate evidence that may prove useful to inform interventions and preparations for public health authorities.

By taking into account vaccination rates from December, 2020 to July, 2021 in Costa Rica, as well as levels of vaccine acceptance, our results suggest that reducing the daily application of first doses by an average of $57\%$ during a 5 month time-frame, Covid-19 related hospitalizations would experience an increase of $25.85\%$ and $22.66\%$, in the scenarios of pre-Delta and Delta circulation, which translates in a $7.9\%$ and $6.75\%$ increase in mortality by the end of the study period. These results go in hand with previous studies that evidence the reduction in hospitalization and mortality with high efficacy mass vaccination campaigns^28,45–47^.

Regarding the introduction of the Delta variant, we predicted that its entrance and gradual dominance in Costa Rica may increase cumulative hospitalizations by $36.79\%$ and $33.30\%$, and deaths by $9.3\%$ and $9.04\%$, in both the high and low vaccination scenarios. Although these figures are significant, they may contrast with some of the most abrupt increases seen in other jurisdictions, such as Israel or the UK, which may have several explanations^48,49^. First, we assumed an introduction of the Delta on July 2021, thus, in our scenarios, Delta would not become relevant before August. In all four scenarios, Covid-19 hospitalizations and ICU admissions were projected to increase by the end of June 2021, regardless of the presence of Delta. This assumption is relevant because the magnitude of the projected outbreak depends not only in changes of transmissible and immune evasion of the virus, but also on the date of introduction of the variant, changes in social behavior, and vaccination coverage achieved at the time Delta becomes the dominants strain. Therefore, in the context of “delayed” Delta introduction and high vaccination coverage, even in the low vaccination rate scenario, our assumptions may have led to an optimistic result.

All scenarios maintained current levels of NPI, including personal protection measures, mobility, and social gathering restrictions, which were only relaxed when $60\%$ of the population had a complete vaccination scheme, assuming changes in social behaviour and policy due to high levels of confidence. In a context where increasing signs of pandemic fatigue among the population^52^ are arising, maintaining these levels of compliance represents a challenge for public health authorities in the path for health, social and, economic recovery. Several of the sharp increases in cases seen in countries with high vaccination rates following the introduction of the Delta variant are in the context of NPI being relaxed ^48^.

Costa Rica is characterized for its near to universal health care coverage and relatively high historic investment in health promotion and prevention campaigns^53,54^. These fundamental factors have provided a relative protection against Covid-19 throughout the pandemic, flattening epidemiological curves and mitigating the effects of the pandemic in the country.

The impact of a new variant can change geographically. Some previous variants of interest have made dramatic appearances in early locations and diminishing its impact in other jurisdictions where they reach dominance at a slower pace competing for dominance with other variants. This was for example the case of the Gamma variant outside South America.

Results from the model also allowed us to estimate the increase in hospital care costs in each scenario, taking into account the base cost for hospital and ICU beds as reported by CCSS^36^. A slower vaccine administration pace translated into a total increase of 43,636,880 USD in base hospital care for the public health care system in the presence of the Delta variant. As the cost of diagnostics, specialized care and other procedures are not considered, the increase in costs for the social security in Costa Rica is expected to be substantially higher. Moreover, although a minority, several of these patients are managed in private hospitals, where the cost of base care is substantially higher. Additionally, if we take into account Costa Rica’s near universal health care system, health care cost for the CCSS is expected to be substantially lower compared to other countries with more fragmented, unequal and limited health care coverage. It is worth noting that financing for the universal public health care system in Costa Rica comes from three sources: employers, workers, and the State, being the monthly social security contributions from its residents the main source of income. According to data from CCSS, income from social contributions from January 2020 to October 2020 decreased by 274,497,894 USD^55^, due to loss of jobs and income from the population, making our results even more relevant to consider for the allocation of resources within an already constrained system.

There are limitations to using these mathematical and statistical tools that mostly lie in the model assumptions. For example, in this study, vaccines are applied randomly to susceptible and recovered individuals, regardless of their geographic location. Moreover, it is likely that the uptake of the vaccine is regionally correlated^56^, leading to foci of high susceptibility in the population, which could act as small-scale outbreak sites reducing the effect of the immunity of the population^57^. Furthermore, the absorption of the vaccine may vary over time and geographically as the perception of risk changes^58^. On the other hand, this assumption ignores that pre-symptomatic or asymptomatic people can receive the vaccine without knowing that they are carriers of the virus. Another relevant assumptions is related to the period that is needed to reach immunity after vaccination. This value is included only implicitly in the model, through the reduction of the percentages of protection against hospitalization. Studies report that protection after first dose begin approximately 12 days after first dose^59^ and that it takes an estimated 14 days to reach maximum level of protection after second dose^60,61^. This period could become relevant when a high percentage of people is immunized and slightly shift our curves to the right.

The use of network models to forecast the effects of different vaccine strategies is a valuable tool for decision-makers. Within a global scenario of considerable disparities in access to Covid-19 vaccines ^62^, middle-income countries, such as Costa Rica, are starting to see significant increases in the supply of vaccines, now that worldwide production has strengthened and the wealthiest nations have secured their supplies. However, we are now facing some of the same threats of vaccine hesitation among younger and healthier population groups. Furthermore, several countries have started to provide booster shots, which may slow down vaccine supplies worldwide again. Our results provide evidence of the impact that implementing successful vaccination campaigns may have on morbidity and mortality at the population level. We also provide initial evidence of the potential cost-effectiveness of such interventions, although further economic research is needed.

## Data Availability

All the codes and files necessary to reproduce the results presented in this document are available in the GitHub repository, https://github.com/EpiMEC/NetworkModel_VaccinationScenarios
